# Synergistic Antifungal Activity of Zinc Pyrithione and Nystatin against Multi-Drug-Resistant *Candida* (*Candidozyma*) *auris*: Evidence from *In Vitro* and *In Vivo* Models

**DOI:** 10.1021/acsinfecdis.5c00822

**Published:** 2026-02-26

**Authors:** Larissa Rodrigues Pimentel, Fabiola Lucini, Ludmilla Cardoso Coferri, Yasmim Isabel Retore, Julia Pimentel Arantes, Cleison da Rocha Leite, Adriana Araújo de Almeida-Apolonio, Carlos Reinier Garcia Cardoso, Alex Polatto Carvalho, Fabricio Fagundes Pereira, Simone Simionatto, Kelly Mari Pires de Oliveira, Luana Rossato

**Affiliations:** † Health Sciences Research Laboratory, Federal University of Grande Dourados, Dourados, Mato Grosso do Sul 79804-970, Brazil; ‡ Microbiological Assays Laboratory, Federal University of Grande Dourados, Dourados, Mato Grosso do Sul 79804-970, Brazil; § Insect Biological Control Laboratory, Federal University of Grande Dourados, Dourados, Mato Grosso do Sul 79804-970, Brazil

**Keywords:** Biofilm, *Candida auris*, fungal infection, repurposing, synergy

## Abstract

Candida (*Candidozyma*) *auris* is a multidrug-resistant fungal pathogen that presents a growing global health concern due to its resistance to conventional antifungals. This study evaluated the antifungal potential of zinc pyrithione (ZnPT) and nystatin (NYS), both individually and in combination, against *C. auris*. Minimum inhibitory and fungicidal concentrations were determined, alongside assays for biofilm inhibition and eradication, including tests on porcine skin. Mechanistic evaluations included assessments of cell membrane integrity, efflux pump inhibition, and sorbitol protection. Safety was analyzed through hemocompatibility, the Ames test, and acute toxicity in *Tenebrio molitor* larvae. ZnPT + NYS combination had a synergistic antifungal effect, effectively inhibiting biofilm formation and increasing membrane permeability, as evidenced by protein leakage. No nucleotide leakage or mutagenic effects were observed, indicating low genotoxic risk. While ZnPT alone exhibited toxicity in *T. molitor*, the combination remained within safe limits. Overall, the ZnPT + NYS combination demonstrated strong antifungal and antibiofilm activity against *C. auris*, with favorable safety outcomes. These findings support further investigation into its clinical potential as a treatment strategy against this emerging pathogen.

## Graphical abstract

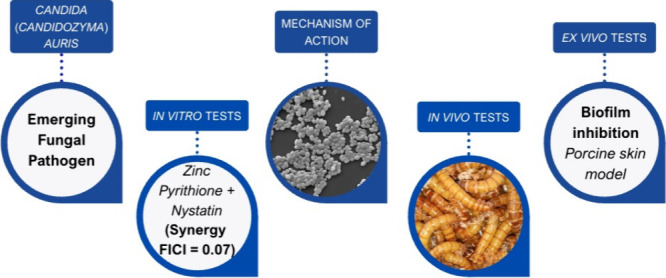

*Candida* (*Candidozyma*) *auris* has emerged as a multidrug-resistant pathogen, causing hospital outbreaks worldwide.^1–3^ Since its first identification in 2009,^4^ *C. auris* infections have risen sharply worldwide, posing a significant threat to public health.^3^ Those most at risk include patients who have recently undergone surgery, used invasive medical devices, received prolonged antibiotic treatments, had extended stays in healthcare settings, or have compromised immune systems.^5^

The spread of *C. auris* poses a public health challenge due to its persistence on surfaces,^6^ and resistance to hospital disinfectants,^7^ complicating infection control. Misidentification by traditional diagnostics further delays treatment and facilitates its spread.^8^

*Candida auris* causes invasive infections, impacting the bloodstream, wounds, and ears, with mortality rates reported between 28% and 56%.^8,9^ Its exceptional persistence within patients and on inanimate surfaces in healthcare environments makes it particularly challenging to control and eradicate.^10^ While early reports in 2009 indicated that *C. auris* was fully susceptible to available antifungals,^4^ resistance to the azole antifungal fluconazole was observed by 2011.^11^ Most *C. auris* isolates resist at least two of the three main antifungal classes, highlighting the need for new therapies.^12^ One particularly challenging aspect is the pathogen’s ability to form biofilms.^13^ Biofilms provide a protective barrier for the yeast, significantly enhancing its ability to withstand antifungal agents and survive in harsh environments.^14^

Considering this, new strategies targeting biofilm-associated resistance are essential to improve treatment outcomes in fungal infections. Fungal skin infections represent a persistent clinical challenge, particularly due to the protective nature of biofilms that enhance microbial resistance and reduce the efficacy of conventional treatments. Addressing this challenge, the application of zinc pyrithione (ZnPT) in topical formulations presents a direct and localized therapeutic approach. ZnPT is a broad-spectrum antifungal and antimicrobial agent commonly used in dermatological products, including treatments for seborrheic dermatitis, psoriasis, and other dermatoses.^15^

Drug repurposing is an increasingly valuable strategy for rapidly identifying treatments for emerging infections across diverse pathogens.^16,17^ To evaluate the therapeutic potential of this approach against *C. auris*, this study combines *in vitro*, *ex vivo*, and *in vivo* models to assess the efficacy and safety of ZnPT in combination with nystatin (NYS). The *ex vivo* model using porcine skin offers a physiologically relevant substrate for studying biofilm adhesion and treatment efficacy under conditions that closely mimic human skin.^18^ In parallel, *Tenebrio molitor*, a coleopteran insect of the *Tenebrionidae* family, was employed as an *in vivo* infection model. This insect has a well-characterized innate immune system, including antimicrobial peptides and hemocyte-mediated responses such as phagocytosis and encapsulation. It has been validated for the study of fungal pathogens, including *C. albicans*, *Cryptococcus neoformans*, and *C. auris*,^19^ offering an ethical, cost-effective, and biologically relevant platform for toxicity and survival assays.

This study investigates the *in vitro*, *ex vivo*, and *in vivo* efficacy of ZnPT in combination with NYS as a potential alternative therapy against multidrug-resistant *C. auris*.

## Results

### Antifungal Susceptibility Testing

*Candida auris* isolate used in this study (150/23) was previously identified as multidrug-resistant, exhibiting reduced susceptibility to at least two major classes of antifungal agents. This resistance profile reflects the challenging nature of treating *C. auris* infections in clinical settings and highlights the urgency in developing therapeutic alternatives.

ZnPT showed an MIC of 64 mg/L, while NYS had an MIC of 32 mg/L for the *C. auris* strain (Table 1). Fungicidal activity was confirmed at these same concentrations: 64 mg/L for ZnPT and 32 mg/L for NYS (Table 1). MIC and MFC values for the *C. auris* CBS strain, used as a control, are also provided in Table 1. The combination of ZnPT and NYS demonstrated a synergistic effect against the *C. auris* strain. The combination resulted in a 64-fold reduction in the concentration of ZnPT and a 16-fold reduction for NYS, yielding a FICI value of 0.07 (Table 1). In addition, a panel of additional clinical *C. auris* isolates available in our laboratory collection was included in the initial screening to broaden the assessment of the ZnPT–NYS interaction (Table 1). This synergistic efficacy was confirmed using Synergy Finder Software (Figure S1).

**Table 1:** Minimum Inhibitory Concentrations (MICs) and Minimum Fungicidal Concentrations (MFCs) of ZnPT and NYS against *C*. *auris* Strains^a^

	MIC (mg/L)				MFC (mg/L)		Checkboard	
Drug	ZnPT (i)	ZnPT (c)	NYS (i)	NYS (c)	ZnPT	NYS	FICI	Interaction
C. auris (150/23)	64	1	32	2	64	32	0.07	SYN
C. auris (150/23)	64	4	64	8	64	64	0.18	SYN
C. auris (151/23)	64	2	32	8	64	32	0.28	SYN
C. auris (153/23)	64	4	64	8	64	64	0.18	SYN
C. auris (158/23)	128	4	32	4	128	32	0.15	SYN
C. auris CBS 10913	32	-	8	-	32	8	-	-

a MIC: minimum inhibitory concentration, MFC: minimum fungicidal concentration. ZnPT: zinc pyrithione, NYS: nystatin. (i): isolate. (c): combinate. FICI (fractional inhibitory concentration index) is used to measure the interaction between the tested combinations. FICI interpretation corresponded to the following definitions: synergism (SYN), FICI ≤ 0.5; no interaction, FICI > 0.5 and ≤ 4; and antagonism (ANT), FICI > 4. MIC, MFC, and FICI values were determined in three independent biological replicates.

### Fungal Inhibition Assay

The efficacy of ZnPT, both as a standalone treatment and in combination with NYS, in inhibiting *C. auris* growth revealed a reduction in fungal growth after 12 h of incubation compared to the positive control. No statistically significant differences were observed among the treatments, except for a significant difference between the positive control (*C. auris* alone) and BZK (Figure 1). The other treatments did not exhibit significant differences, either when compared to one another or to the positive control.

**Figure 1:**
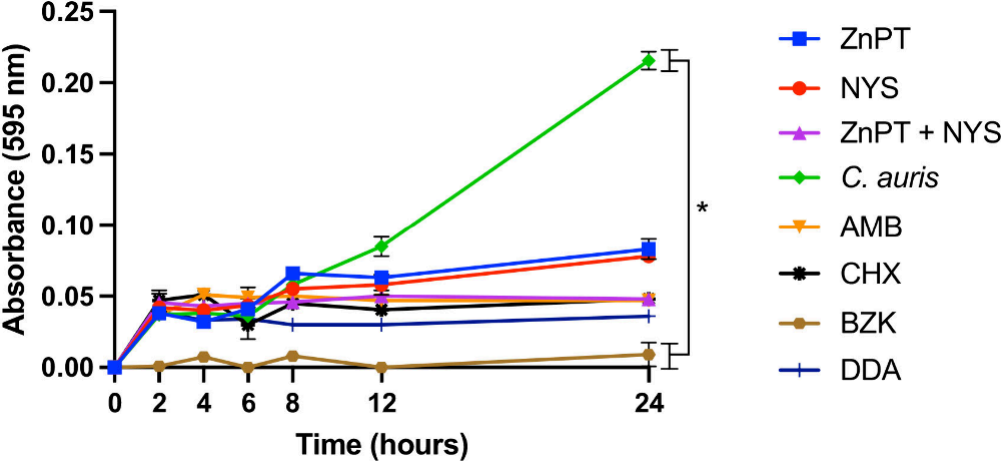
*In vitro* fungal growth inhibition of *C. auris* by ZnPT and NYS. The test was conducted at concentrations of ZnPT (at MIC 64 mg/L), NYS (at MIC 32 mg/L), ZnPT (at FIC 1 mg/L) + NYS (at FIC 2 mg/L). Treatments included comparative controls such as AMB, CHX, BZK, and DDA. ZnPT: zinc pyrithione, NYS: nystatin, AMB: amphotericin B, CHX: chlorhexidine, BZK: benzalkonium chloride, DDA: didecyldimethylammonium chloride. Data represent mean ± SD from three independent biological experiments. Statistical analysis was performed using one-way ANOVA with Tukey’s multiple-comparison test. * *p* < 0.05.

### Antibiofilm Activity

Antibiofilm activity assay was conducted on *C. auris* using ZnPT at 64 mg/L, NYS at 32 mg/L, and a combination of ZnPT (1 mg/L) + NYS (2 mg/L). The combination of ZnPT + NYS demonstrated superior efficacy, achieving an antibiofilm activity rate of 50.39%. ZnPT alone exhibited an activity rate of 31.70%, while nystatin showed 20.45%. These results highlight that the ZnPT + NYS combination is significantly more effective in inhibiting biofilm formation compared to either compound used individually (Figure S2).

### Effect of ZnPT and NYS on Inhibiting *C*. *auris* Biofilm Adhesion to Porcine Skin

The combination of ZnPT + NYS inhibited *C. auris* biofilm formation on porcine skin over time, with inhibition rates exceeding 95% across the evaluated time points. At exposure times of 15 and 45 min, the ZnPT + NYS treatment achieved inhibition rates of approximately 96% and 98.2%, respectively. At subsequent times of 4 and 24 h, the inhibition rate reached over 99%. Statistical analysis revealed no significant difference between the ZnPT + NYS and CHX treatments (*p* = 0.5397; Figure 2).

**Figure 2:**
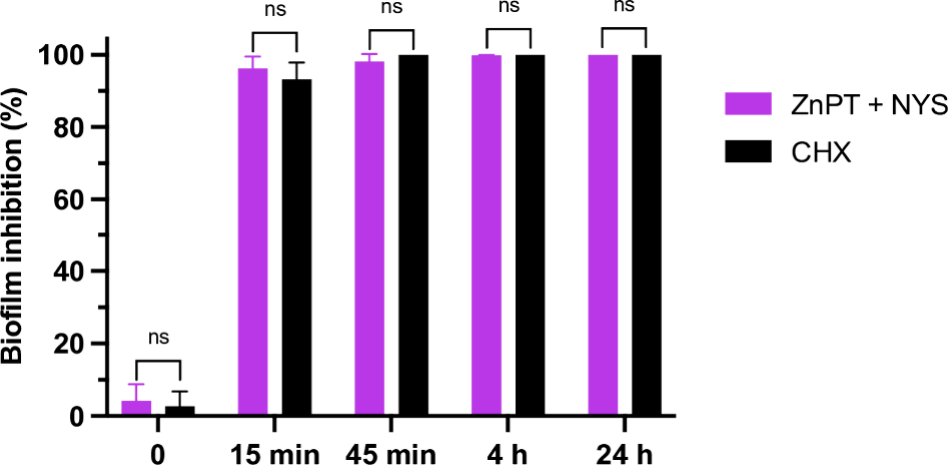
*Ex vivo* inhibition of *C. auris* biofilm adhesion to porcine skin by ZnPT and NYS. Porcine skin discs were inoculated with *C. auris* and treated with ZnPT + NYS or CHX. Biofilm formation was quantified by CFU enumeration after vortexing. ZnPT: zinc pyrithione; NYS: nystatin; CHX: chlorhexidine. Data represent mean ± SD from three independent biological experiments. Statistical analysis was performed using the Mann–Whitney U test.

### Biofilm Eradication Assay

A biofilm eradication assay was performed on *C. auris* using ZnPT at 64 mg/L, NYS at 32 mg/L, and a combination of ZnPT (1 mg/L) + NYS (2 mg/L), with CHX, BZK, and DDA included as comparative disinfectants. The ZnPT + NYS combination achieved biofilm eradication rates of 85.21% at 15 min and 85.96% at 45 min. ZnPT alone exhibited minimal biofilm eradication at both evaluated exposure times. In contrast, nystatin exhibited a biofilm eradication rate of 41.70% at 15 min and 59.14% at 45 min. BZK and CHX showed eradication rates of 70.27% and 59.21%, respectively, at both 15 min (Figure 3).

**Figure 3:**
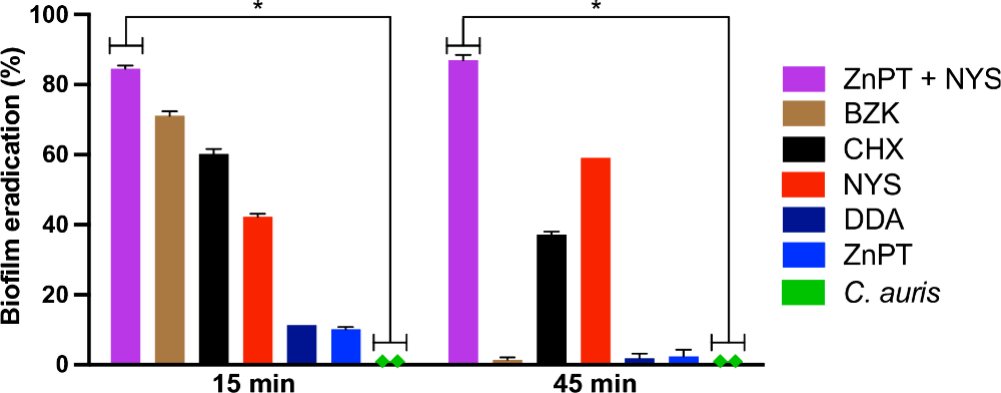
Biofilm eradication on catheter surfaces. Double-lumen central venous catheters were incubated with *C. auris* to allow biofilm formation, followed by exposure to ZnPT, NYS, and their combination. The test was conducted at concentrations of ZnPT (at MIC 64 mg/L), NYS (at MIC 32 mg/L), and ZnPT (at FIC 1 mg/L) + NYS (at FIC 2 mg/L). Eradication efficacy was assessed by CFU counting after biofilm dislodgement. Treatments included comparative controls such as CHX, BZK, and DDA. ZnPT: zinc pyrithione; NYS: nystatin; CHX: chlorhexidine; BZK: benzalkonium chloride; DDA: didecyldimethylammonium chloride. Data represent mean ± SD from three independent biological experiments. Statistical analysis was performed using one-way ANOVA with Tukey’s multiple-comparison test. * *p* < 0.05.

### Sorbitol Protection Assay

The effects of ZnPT, NYS, and their combination on *C. auris* in the sorbitol protection assay suggest a possible involvement of cell envelope or cell-wall stress, as indicated by increased MIC values in the presence of sorbitol. An increase in MIC under osmotic stabilization conditions is commonly associated with alterations in cell-wall integrity. Thus, these findings indicate that cell-wall stress may contribute to the antifungal activity of these compounds (Supplementary Table S1).

### Efflux Pump Inhibition Assay

*C. auris* cells were exposed to treatments at concentrations of 64 mg/L of ZnPT, 32 mg/L of NYS, and 1 mg/L of ZnPT + 2 mg/L of NYS, with and without the addition of promethazine (128 mg/L), an established efflux pump inhibitor. The presence of promethazine resulted in reduced MIC values for ZnPT and NYS, indicating that efflux-related processes may contribute to antifungal susceptibility under these conditions (Supplementary Table S2). Consistent with these observations, the Rhodamine 6G assay indicated altered efflux-related activity in treated cells, supporting a potential contribution of efflux modulation to the enhanced antifungal effects observed in susceptibility assays (Figure 4).

**Figure 4:**
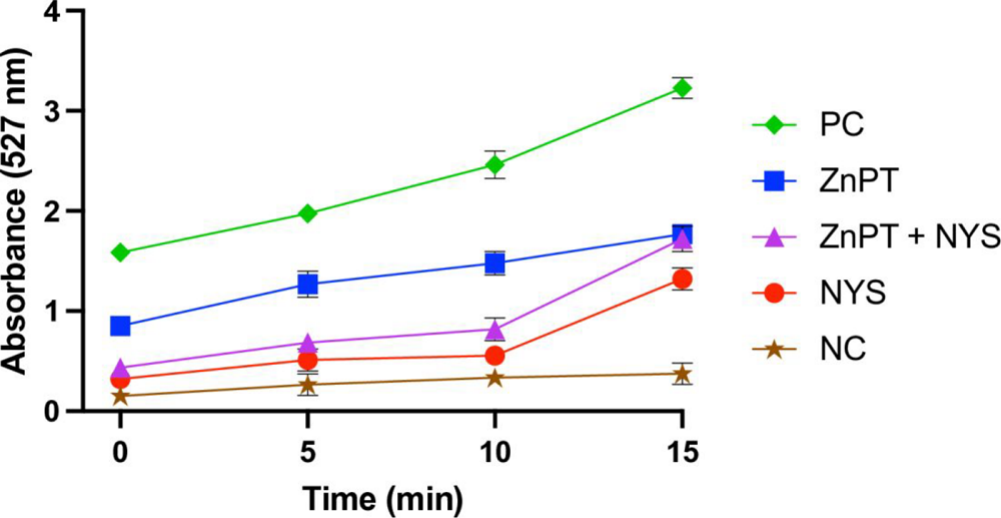
Rhodamine 6G extracellular efflux assay. Extracellular R6G fluorescence was monitored over time following glucose addition. Data represent mean ± SD from three independent biological experiments. NC: negative control (glucose-free condition); PC: positive control (glucose-supplemented condition).

### Alteration of Cell Membrane Permeability

A trend toward increased protein leakage was observed after treatments with ZnPT and NYS, both individually and in combination, compared to AMB. However, statistical analyses revealed no significant differences between the treatments and the positive control (*C. auris* alone). The results indicated that ZnPT alone showed minimal protein leakage after 2 h compared to the combination treatment, which caused greater disruption to the fungal cell membrane, leading to higher protein concentrations in the supernatant over time. After 4 h, treatments with ZnPT and NYS alone resulted in protein leakage levels of 38.33 mg/L and 70.83 mg/L, respectively, while the combination showed a level of 28.33 mg/L (Figure S3).

### Nucleotide Leakage

Nucleotide leakage was assessed by measuring absorbance at 260 nm in the supernatants after exposure to ZnPT (MIC 64 mg/L), NYS (MIC 32 mg/L), and their combination (ZnPT at FIC 1 mg/L + NYS at FIC 2 mg/L). AMB was used as a positive control for nucleotide leakage, and untreated *C. auris* served as the negative control. No significant differences in nucleotide leakage were observed between the treatments (ZnPT, NYS, and combination) and the positive control (AMB) at the 2-h time point. At 4 h, the combination treatment caused 0.75 mg/L nucleotide leakage, compared to 0.95 mg/L for AMB. ZnPT alone caused minimal nucleotide leakage (0.04 mg/L at 2 h), while NYS alone showed no measurable release at 2 h. Although a trend toward increased nucleotide leakage was observed with the combination treatment at 4 h, statistical analysis revealed no significant differences between treatments. *ns* indicates no statistically significant difference compared to the positive control (*C. auris* alone) (Figure S4).

### Scanning Electron Microscopy (SEM)

Cells of *C. auris* treated with ZnPT (64 mg/L) exhibited mild morphological alterations, characterized by detectable surface damage, including surface roughening (Figure 5a). Treatment with NYS alone (32 mg/L) induced subtle morphological changes in a subset of cells, with limited surface irregularities observed (Figure 5b). Similarly, exposure to the ZnPT (1 mg/L) + NYS (2 μg/mL) combination resulted in only mild morphological alterations, with slight surface irregularities observed (Figure 5c). Untreated *C. auris* cells displayed smooth and intact surfaces (Figure 5d).

**Figure 5:**
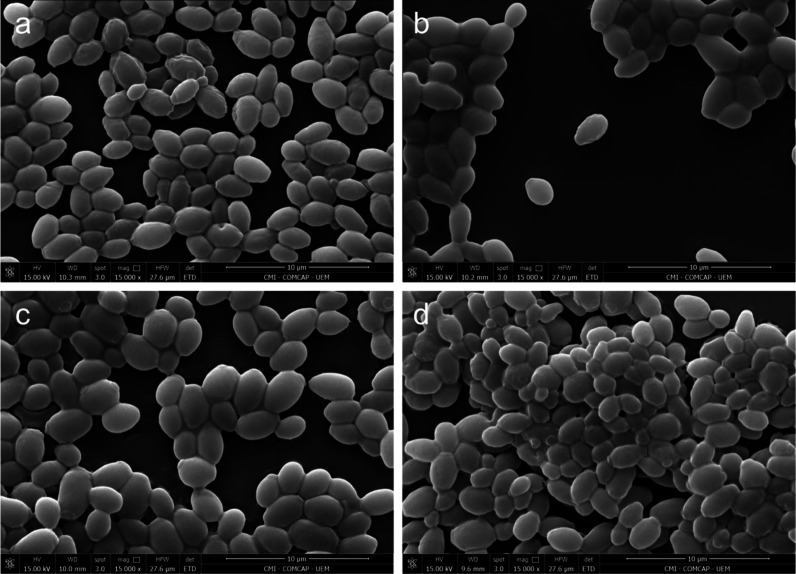
Scanning electron microscopy images of *C. auris* subjected to different treatments. (a) ZnPT alone (64 mg/L). (b) NYS alone (32 mg/L). (c) Combination both (1 mg/L ZnPT + 2 mg/L NYS). (d) Untreated control.

### Hemolysis Assay

At isolated concentrations of 64 mg/L for ZnPT and 32 mg/L for NYS, neither compound showed evidence of inducing hemolysis, nor did the combination of ZnPT (1 mg/L) + NYS (2 mg/L). These results indicate that both ZnPT and NYS are hemocompatible and nonhemolytic (Figure 6a).

**Figure 6:**
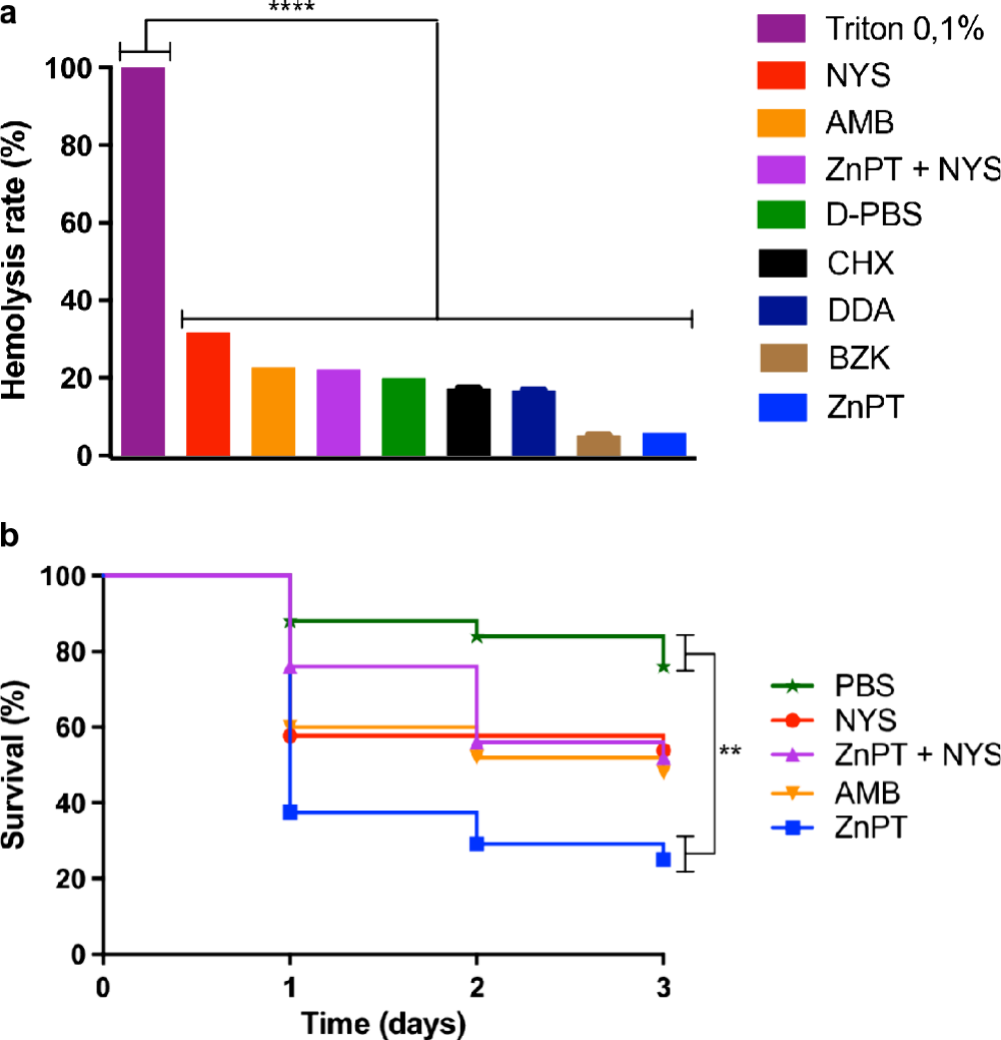
*In vitro* hemocompatibility and *in vivo* acute toxicity of ZnPT and NYS. (a) Treatments included ZnPT (64 mg/L), NYS (32 mg/L), and the combination (1 mg/L ZnPT + 2 mg/L NYS). D-PBS was used as the negative control, and 0.1% Triton X-100 served as the positive control. Statistical analysis was performed using one-way ANOVA with Tukey’s multiple-comparison test. (b) *In vivo* acute toxicity of ZnPT and NYS using the *T. molitor* larvae model. Survival was analyzed using the log-rank (Mantel–Cox) test. ZnPT: zinc pyrithione, NYS: nystatin, AMB: amphotericin B, CHX: chlorhexidine, BZK: benzalkonium chloride, DDA: didecyldimethylammonium chloride. Data represent mean ± SD from three independent biological experiments. **** *p* < 0.0001, ** *p* < 0.01.

### Acute Toxicity Tests Using *T*. *molitor*

Acute toxicity assays on *T. molitor* revealed significant differences in survival among the treatments. Comparisons were conducted using ZnPT at 64 mg/L, NYS at 32 mg/L, and their combination of ZnPT at 1 mg/L with NYS at 2 mg/L, based on the concentrations determined from the MIC tests. Statistically, only ZnPT alone showed a significant difference when compared to the negative control (PBS), suggesting that this treatment may be considered toxic in the *T. molitor* model (Figure 6b).

### Ames Test

Results were analyzed using Salanal software, which indicated no significant mutagenicity for ZnPT + NYS at the tested concentrations, supporting a low risk of mutagenic effects (Table 2).

**Table 2:** Mutagenic Activity of ZnPT Combined with NYS, Expressed As the Mean Number of Revertant Colonies/Plate ± Standard Deviation and the Mutagenicity Index against *S.* *typhimurium* Strains TA98 and TA100 in the Absence (-S9) and Presence (+S9) of Metabolic Activation

	TA98		TA100	
μg/plate	–S9	+S9	–S9	+S9
**0** ^b^	35 ± 2	33 ± 4	155 ± 7	102 ± 5
**2**	33 ± 1 (1.0)	29 ± 3 (0.9)	143 ± 5 (0.9)	128 ± 2 (1.2)[Table-fn t2fn6]
**5**	35 ± 6 (1.0)	27 ± 2 (0.8)	131 ± 5 (0.8)	129 ± 4 (1.3)[Table-fn t2fn6]
**15**	35 ± 6 (1.0)	28 ± 2 (0.8)	131 ± 3 (0.8)	139 ± 2 (1.4)[Table-fn t2fn6]
**50**	32 ± 2 (0.9)	30 ± 2 (0.9)	130 ± 5 (0.8)	137 ± 2 (1.3)[Table-fn t2fn6]
**150**	41 ± 2 (1.2)	30 ± 2 (0.9)	139 ± 6 (0.9)	140 ± 9 (1.4)^e^
**C+**	260 ± 9^c^	293 ± 7^d^	677 ± 9^c^	708 ± 7^d^

a Negative control: DMSO.

b Positive control (C+): 4-Nitrophenylenediamine (10 μg/plate).

c Positive control (C+): 2AA-Aminoanthracene (1.5 μg/plate).

d Significant difference (ANOVA): *p* < 0.05.

e Significant difference (ANOVA): *p* < 0.01.

## Discussion

Since its discovery over a decade ago, *C. auris* has emerged as a significant nosocomial pathogen with unique skin tropism, enabling persistent colonization and widespread hospital outbreaks. This challenges current treatment strategies and underscores the urgent need for new antifungal solutions.^20,21^ ZnPT is a zinc coordination complex approved by the United States Food and Drug Administration (FDA) as an additive for treating seborrheic dermatitis and dandruff, owing to its well-documented antimicrobial and antifungal properties.^15^ NYS, a well-established antifungal, was studied individually and in combination with ZnPT.

Although ZnPT, alone or with NYS, reduced fungal growth, differences were not statistically significant versus AMB, CHX, or DDA. Still, lower absorbance versus the yeast-only control suggests inhibited proliferation. After 24 h, growth did not resume, indicating fungicidal activity. ZnPT likely acts by increasing intracellular copper, damaging iron–sulfur clusters,^22^ while NYS binds ergosterol, forming pores and causing cell death.^23^

The apparent difference in the time required to observe antifungal effects in planktonic growth and biofilm assays reflects the different parameters measured by these models. Planktonic growth assays evaluate cumulative effects on cell proliferation, whereas biofilm assays examine treatment effects on preformed adherent biomass over defined exposure periods.

Our biofilm inhibition tests, including those conducted on porcine skin, demonstrated that the combination of ZnPT and NYS significantly reduced *C. auris* biofilm formation. Since biofilms drive antifungal resistance in *C. auris*, this has important clinical implications.^24^ The ability of ZnPT + NYS to inhibit biofilm formation on a model simulating human skin underscores its potential as an effective topical treatment against *C. auris*, particularly in preventing skin colonization in healthcare environments. Studies exploring the use of NYS and ZnPT for treating *C. auris* infections in porcine skin models are limited. However, porcine skin models have been used to compare fungal colonization across different *Candida* species, biofilm formation, and the effectiveness of various treatments.^18,25^

ZnPT + NYS outperformed the individual treatments in *C. auris* biofilm eradication, achieving high eradication rates (∼85–86%) at both evaluated exposure times. This highlights its efficacy in disrupting biofilms, crucial for managing persistent infections.^26^ Notably, ZnPT + NYS also demonstrated greater effectiveness than CHX under the tested conditions.

Among antifungal combinations with potential clinical applications, the synergy observed between ZnPT and NYS surpasses that of other antifungal pairings commonly considered for clinical use.^27,28^ Although pairings such as NYS + CHX are occasionally employed in topical applications, evidence supporting genuine pharmacodynamic synergy is limited.^23^ Some studies have reported a lack of enhanced efficacy and potential chemical instability when these agents are combined, possibly due to pH shifts and surfactant interactions that compromise nystatin’s integrity and bioactivity. Similarly, previous work has shown that ZnPT can be inactivated by cationic surfactants like CHX, which interfere with its mechanism of action through physicochemical interactions that disrupt metal–ligand complexation and membrane activity.^29^ In contrast, the ZnPT + NYS combination demonstrated not only significant reductions in MICs for both agents. The combination showed no hemolytic or mutagenic activity and low toxicity in *T. molitor*, supporting its topical potential. Taken together, these findings suggest that ZnPT + NYS offers a more robust and pharmacologically sound alternative than either ZnPT + CHX or NYS + CHX, and may represent a promising strategy for the treatment of drug-resistant *C. auris* infections.

Fungal cells with damaged walls cannot regulate osmotic pressure and fail to grow without sorbitol. When added, sorbitol restores balance, allowing growth despite wall damage, helping identify cell wall inhibitors by increasing MIC values in its presence.^30^

The reduction in MIC values observed in the presence of promethazine suggests that efflux-related processes may contribute to the antifungal susceptibility of *C. auris* to ZnPT and NYS. Consistent with this observation, the Rhodamine 6G assay provided complementary functional evidence of altered efflux activity in treated cells, supporting a potential role of efflux modulation under these conditions. Nystatin exerts its antifungal activity primarily through interaction with ergosterol in the fungal cell membrane, leading to pore formation and membrane disruption,^31^ whereas ZnPT affects intracellular metal homeostasis and essential cellular processes.^22^ Although these mechanisms are not classically described as efflux-dependent, modulation of efflux-related processes may influence intracellular exposure to these compounds and thereby contribute to the enhanced antifungal activity observed when they are used alone or in combination.

ZnPT likely disrupts fungal membrane integrity by targeting phospholipid heads, causing loss of cellular components and impaired function.^22^ NYS, on the other hand, exerts its antifungal action by binding to sterols within the fungal cell membrane, altering membrane permeability and inducing leakage of cytoplasmic contents.^32^ Notably, when ZnPT and NYS are combined, the degree of protein extravasation surpasses that observed with amphotericin B, a well-established membrane- permeability antifungal.^33^

Nucleotide leakage analysis revealed that the ZnPT + NYS combination caused only limited release of intracellular nucleotides, reaching 0.75 mg/L after 4 h—lower than the 0.95 mg/L observed with the positive control, AMB. ZnPT caused minimal leakage (0.04 mg/L at 2 h), and NYS alone caused no measurable release. Their combination disrupted membranes without excessive cytoplasmic loss. Similarly, in a study with *Centrosema coriaceum* extract, NYS increased 260 nm absorbance over 5 h, indicating nucleotide leakage. This effect is consistent with nystatin’s known mechanism of binding to membrane sterols, increasing permeability and promoting cytoplasmic leakage, as previously reported in *C. glabrata*.^34^

Consistent with these permeability data, SEM revealed only mild morphological alterations in *C. auris* cells treated with ZnPT, NYS, or their combination, primarily manifested as slight surface roughening. These observations support the notion that the treatments induce subtle perturbations of the fungal cell envelope without extensive structural damage.

Additionally, our study demonstrated no hemolytic activity for either compound, with the combination of ZnPT and NYS showing an even lower hemolysis rate compared to the individual agents. The hemolytic activity of NYS in some formulations was evaluated and found to be influenced by its aggregation state, with reduced hemolytic activity under optimized conditions, indicating compatibility with red blood cells.^35^ However, nystatin is known to exhibit toxicity at higher concentrations, particularly when administered systemically, due to its mechanism of action that involves binding to sterols in cell membranes, which can also affect mammalian cells.^36^ Its use is mainly restricted to topical applications to avoid systemic toxicity. ZnPT, however, may cause hemolysis at high concentrations, requiring careful dosage assessment for safe use.^37^

The Ames test showed no significant mutagenicity for ZnPT + NYS, indicating low genetic risk and supporting its clinical potential. While ZnPT’s toxicity has been reviewed, its effects on long-term exposure, metabolism, and bioaccumulation in clinical contexts remain unclear.^38^ Acute toxicity tests in *T. molitor* showed ZnPT’s toxicity, while ZnPT + NYS remained within a safe range. This model is valuable for studying antifungals due to its conserved immune system.^19^ A study on Nyotran, a liposomal nystatin formulation, found no significant genotoxic or mutagenic effects in animal models, supporting its therapeutic safety.^36^

## Conclusions

This study demonstrates that the combination of ZnPT and NYS is effective both *in vitro* and *in vivo* against multidrug-resistant *C. auris*, supporting its potential as a topical therapeutic option. The formulation showed strong antifungal and antibiofilm activity, with low toxicity and no mutagenic effects. However, the limited number of isolates tested is a constraint, as it may not capture the full genetic and resistance variability of *C. auris* worldwide. Further studies using a broader range of strains, extended toxicity assessments, and clinical validation are needed to confirm its safety and therapeutic value in real-world settings.

## Materials and Methods

### Strain and Culture Conditions

The fungal strain used in this study was obtained from the Mycotheque of the Núcleo de Estudos em Micologia Médica Aplicada (NEMMA) at the Health Sciences Research Laboratory (LPCS), Federal University of Grande Dourados. We utilized a previously identified multidrug-resistant isolate of *C. auris* (150/23), belonging to clade IV, identified during an outbreak in Venezuela and previously sequenced.^7,9^ In addition, other clinical *C. auris* isolates available in our laboratory collection were included in the initial susceptibility screening. For testing, the strain was transferred onto freshly prepared Sabouraud Dextrose Agar (SDA) and incubated for 24 h at 37 °C. This study was approved by the local institutional review board with approval no 048021/2015 (Ethics Committee of Federal University of Grande Dourados).

### Minimum Inhibitory Concentration (MIC)

The Minimum Inhibitory Concentrations (MICs) of NYS and ZnPT were determined following the guidelines from the Clinical Laboratory Standards Institute (CLSI).^39^ Sensitivity profiles for ZnPT and NYS were assessed using dilution ranges of 0.5 to 256 mg/L and 0.12 to 64 mg/L, respectively. Fungal suspensions were prepared at a density of 1–2.5 × 10^3^ CFU/mL. The plates were then incubated at 37 °C for 24 h. MIC was defined as the lowest concentration that visually inhibited 100% of the strain’s growth. To determine the Minimum Fungicidal Concentrations (MFC), 10 μL from each well of the MIC plates was transferred to SDA plates and incubated at 37 °C for 24 h. The *C. auris* reference strain CBS 10913, which belongs to Clade II, was used as an experimental control in this study and is widely used in *C. auris* research. MIC and MFC determinations were performed in three independent biological replicates.

### Checkerboard Assay

The combination of ZnPT and NYS was evaluated using the checkerboard microdilution method.^40^ ZnPT (0.5–256 mg/L) and NYS (2–64 mg/L) were tested in a 96-well plate, with 50 μL of NYS added horizontally and ZnPT vertically. Each well received 100 μL of inoculum (10^3^ cells/mL). Eight wells with RPMI-1640 and MOPS buffer served as sterility controls, while another eight with inoculum acted as positive controls. Plates were incubated at 37 °C for 24 h.

The determination of the Fractional Inhibitory Concentration Index (FICI) of a drug combination is based on Loewe’s additivity and is calculated as follows

$$FICI=\frac{MICofDrug1incombination}{MICofDrug1alone}+\frac{MICofDrug2incombination}{MICofDrug2alone}$$

where MIC of drug 1 in combination and MIC of drug 2 in combination correspond to the MICs of each drug when tested in combination, and MIC of drug 1 alone and MIC of drug 2 alone correspond to the MICs of each drug when tested individually. Drug interactions were defined as synergistic if FICI ≤ 0.5, no interaction if FICI > 0.5 ≤ 4, and antagonistic if FICI > 4.^40^ Experiments were performed in three independent biological replicates.

The SynergyFinder web application was used to perform dose–response analyses and to calculate synergy scores for the ZnPT–NYS combination based on the zero interaction potency (ZIP), Bliss independence, and Loewe additivity models. Synergy surface plots were generated for each model, and confidence intervals were calculated internally by the software. Synergy scores were interpreted as follows: values >10 indicated synergism, values between – 10 and 10 indicated additive effects, and values < – 10 indicated antagonism.

### Inhibition Growth Assay

Cells were standardized to 0.5 McFarland and then diluted to 1–2.5 × 10^3^ cells/mL in RPMI-1640 with MOPS buffer, pH 7.0, at 37 °C. Suspensions were mixed with ZnPT, NYS, their FIC values, AMB (0.5 mg/L as a negative control), and RPMI-1640 with MOPS (positive control). Tubes were incubated at 37 °C, and absorbance at 595 nm was measured at 0, 2, 4, 6, 8, 12, and 24 h to monitor growth kinetics. Growth curves were analyzed for fungicidal effects^12^ using three independent biological replicates. Disinfectant controls, commonly used in hospitals, such as chlorhexidine (CHX), benzalkonium chloride (BZK), and didecyldimethylammonium chloride (DDA), were included.

### Antibiofilm Activity

Biofilm inhibition and eradication assays were conducted using fixed concentrations selected according to the MIC values determined for each compound. *Candida auris* inoculum (1–2.5 × 10^3^ cells/mL) in RPMI-1640 with MOPS was incubated in a 96-well plate at 37 °C for 24 h to form biofilms. After adhesion, nonadherent cells were removed, and biomass was washed, stained with 0.1% crystal violet, and resuspended in 70% ethanol for quantification at 595 nm. Experiments were performed in three independent biological replicates. The percentage of biofilm inhibition for each compound, alone and in combination, was calculated:

$$\%BiofilmInhibition=\left(\frac{ODofthecontrol-ODofthetreatment}{ODofthecontrol}\right)\times 100$$

### Effect of ZnPT and NYS on the Inhibition of *C. auris* Biofilm Adhesion to Porcine Skin

ZnPT + NYS biofilm inhibition was analyzed using a modified *ex vivo* porcine skin model.^25^ Porcine skin samples, sourced from a local supplier, were cut into 12 mm discs using a biopsy punch and decontaminated by immersion in an antibiotic solution (streptomycin 1,000 mg/L and penicillin 1,000 units/mL) for 18 h. The samples were then rinsed with Dulbecco’s Phosphate Buffered Saline (DPBS) and transferred to 12-well plates containing semisolid Dulbecco’s Modified Eagle Medium (DMEM), supplemented with 10% fetal bovine serum.

*Candida auris* was cultured in Sabouraud broth at 37 °C and adjusted to 1–2.5 × 10^3^ cells/mL. Ten microliters of the suspension were applied to porcine skin, followed by ZnPT + NYS (2 mg/L each) or CHX (1%) as a comparison. Samples were incubated at 37 °C for 0, 15, 45 min, 4, and 24 h. After incubation, each skin sample was transferred to a tube containing 10 mL of NaCl solution and vortexed for 10 min to dislodge the cells. Serial dilutions were performed, and the suspensions were plated on SDA. Colony-forming units (CFU) were counted after 24 h of incubation at 37 °C. Experiments were performed in three independent biological replicates. Results were presented as inhibition percentages:

$$\%inhibition=\left(\frac{CFU\text{/}mLpositivecontrol-CFU\text{/}mLtreatment}{CFU\text{/}mLpositivecontrol}\right)\times 100$$

### Biofilm Eradication Assay

*Candida auris* was cultured on SDA for 24 h, then resuspended in peptone water (10^3^ CFU/mL). Double-lumen central venous catheters were immersed in the suspension and incubated at 37 °C for 24 h to form biofilms. Sterility controls used catheters in peptone water without yeast. After incubation, catheters were rinsed to remove nonadherent cells and treated with ZnPT (64 mg/L), NYS (32 mg/L), ZnPT + NYS (1 + 2 mg/L), or peptone water (control) for 15 and 45 min. Biofilm was collected from the catheters using physical agitation. After plating the samples on SDA, they were incubated at 37 °C, and the CFU count was determined. Experiments were performed in three independent biological replicates. Biofilm eradication percentage was calculated with the formula^41^

$$\%\text{Biofilm Eradication}=\left(\frac{\text{CFU of Untreated Biofilm}-\text{CFU of Treated Biofilm}}{\text{CFU of Untreated Biofilm}}\right)\times 100$$

### Sorbitol Protection Assay

Serial microdilutions were prepared in a sterile 96-well microplate containing RPMI-1640 medium with MOPS buffer and enriched with 0.8 M sorbitol. The ZnPT stock solution was diluted to concentrations ranging from 0.5 to 256 mg/L, and NYS concentrations ranged from 0.12 to 64 mg/L, with micafungin (MCF) serving as a positive control. MIC values were determined after incubation at 37 °C for 24 and 48 h.

### Efflux Pump Inhibition Assay

To assess the contribution of efflux-related processes to the activity of ZnPT and NYS, a phenotypic susceptibility assay was performed using promethazine.^42^ ZnPT (0.5 to 256 mg/L) and NYS (0.12 to 64 mg/L) were tested in the presence of a subinhibitory concentrations of promethazine (128 mg/L).

### Rhodamine 6G Extracellular Efflux Assay

Efflux activity was evaluated using a Rhodamine 6G (R6G) extracellular efflux assay.^43^ *Candida auris* cells were grown in YPD broth at 35 °C with shaking (150 rpm) and exposed for 5 h to ZnPT (64 mg/L), NYS (32 mg/L), or the ZnPT (1 mg/L) + NYS (2 mg/L) combination. Cells were then harvested by centrifugation, washed with phosphate-buffered saline (PBS) to remove residual carbon sources, and resuspended in PBS supplemented with 2-deoxy-d-glucose (2 mM) to deplete intracellular ATP. Rhodamine 6G was added to a final concentration of 10 μM, and the suspension was incubated at 35 °C for 2 h to allow dye uptake. Following incubation, cells were collected by centrifugation, washed with sterile distilled water and PBS, and resuspended in PBS. Efflux was initiated by the addition of glucose to a final concentration of 2 mM. At predetermined time points (0, 5, 10, and 15 min), aliquots were collected, centrifuged, and the supernatants were transferred to a 96-well microplate. A glucose-free condition was included as a negative control. Rhodamine 6G fluorescence was measured at room temperature using a fluorescence spectrophotometer at 527 nm.

### Alteration of Cell Membrane Permeability

Cell membrane permeability was evaluated using the BCA Protein Assay Kit. *Candida auris* cells were prepared in sterile distilled water and adjusted to a concentration of 1–2.5 × 10^3^ cells/mL. This cell suspension was combined with ZnPT (MIC 64 mg/L), NYS (MIC 32 mg/L), and ZnPT (MIC 1 mg/L) + NYS (MIC 2 mg/L), then incubated at 37 °C for intervals of 0, 1, 2, 3, and 4 h. After incubation, the samples were centrifuged at 908 g for 5 min at 4 °C. Following this, 25 μL of the supernatant was transferred to a flat-bottom 96-well plate, and 200 μL of BCA working reagent was added to each well. The plate was shaken for 30 s and incubated at 37 °C for 30 min. Absorbance readings were taken at 595 nm after incubation. Background absorbance was corrected by subtracting the mean of control wells. A blank containing only treatment and BCA reagent was also used, and its value subtracted from the corresponding treatment. Protein concentration (mg/L) was calculated using the linear equation from the kit’s calibration curve.^12^ Experiments were performed in three independent biological replicates.

### Nucleotide Leakage

Nucleotide leakage analysis was conducted.^44^ *Candida auris* was grown on SDA at 37 °C for 24 h and suspended in 0.9% saline at 1–2.5 × 10^3^ cells/mL. These suspensions were treated with ZnPT (64 mg/L), NYS (32 mg/L), or a combination of ZnPT (at MIC 1 mg/L) + NYS at MIC 2 mg/L) for different incubation times (samples collected at 0, 1, 2, and 4 h). Following incubation, the samples were centrifuged at 1300 g for 15 min and analyzed at 260 nm. Experiments were performed in three independent biological replicates.

### Scanning Electron Microscopy (SEM)

To examine morphological alterations in *C. auris* induced by ZnPT, NYS, and their combination, scanning electron microscopy (SEM) was employed. *Candida auris* cells (1–2.5 × 10^3^ cells/mL) were exposed to the MICs determined in susceptibility assays and incubated for 24 h at 37 °C. Cells were then harvested by centrifugation at 3000 × g for 5 min at 22 °C, and the supernatants were discarded. The resulting cell pellets were fixed for 5 h at room temperature using a fixation solution prepared in 0.1 M sodium phosphate buffer (pH 7.2).

Following fixation, cells were washed three times with 0.15 M sodium phosphate buffer (pH 7.2), postfixed with 0.2% (w/v) osmium tetroxide, and centrifuged again. Dehydration was carried out through a graded ethanol series (30%, 70%, and three washes with 100% v/v ethanol), with each step lasting 10 min and followed by centrifugation. Samples were subsequently immersed in a 1:1 ethanol/hexamethyldisilazane (HMDS) solution for 10 min, centrifuged, and then washed with 100% HMDS.

The final cell pellet was transferred onto coverslips precoated with 0.1% (w/v) gelatin. After air-drying, coverslips were mounted onto aluminum stubs, sputter-coated with a 20 nm layer of gold using a sputter coater (EMITECH Q150TES, Quorum Technologies, England), and examined by SEM.

### Hemolysis Assay

Sheep erythrocytes isolated from commercially sourced defibrinated sheep blood were diluted 25-fold in sterile PBS, and then 250 μL of the diluted blood was mixed with ZnPT (at MIC 64 mg/L), NYS (at MIC 32 mg/L), and ZnPT (at MIC 1 mg/L) + NYS (at MIC 2 mg/L). PBS and Triton (0.1%, v/v) were used as negative and positive controls, respectively. The samples were incubated at 37 °C for 1 h and subsequently centrifuged at 700 g for 5 min. A 100 μL supernatant from each sample was added to a 96-well plate, and absorbance at 490 nm was measured to calculate the hemolysis ratio.

$$\left(\frac{ODs-ODnc}{ODpc-ODnc}\right)\times 100$$

ODs: OD490 values for samples, ODnc: OD490 values for negative controls, ODpc: OD490 values for positive controls. Experiments were performed in three independent biological replicates.

### Ames Test

The mutagenic potential of the compound ZnPT in combination with NYS was evaluated using the Ames test,^45^ with the *Salmonella enterica* Serovar Typhimurium strains TA98 and TA100, provided by the Toxicology and Genotoxicity department of the São Paulo State Environmental Company (CETESB). The bacterial suspension, at a concentration of 1–2 × 10^9^ cells/mL, was added to tubes containing different concentrations of the test compound (2.0 to 150 mg/L) in the presence of phosphate buffer or S9 fraction. The mutagens 2-aminoanthracene and 4-nitro-o-phenylenediamine were used as positive controls in the presence and absence of metabolic activation, respectively. Dimethyl sulfoxide (DMSO) was used as the negative control. The tubes were preincubated at 37 °C for 90 min.

After preincubation, 2 mL of top agar was added, poured onto glucose minimal agar plates, and incubated at 37 °C for 48–66 h before counting revertant colonies. The mutagenicity index (MI) was calculated using the formula: MI = number of induced revertants/number of spontaneous revertants. The compound was considered to have mutagenic potential when MI ≥ 2.^46^ Concentrations with an MI ≤ 0.7 were considered cytotoxic.^47^

### Acute Toxicity Tests Using the *T. molitor* Model

*Tenebrio molitor* larvae, weighing between 0.110 and 0.200 g, were divided into four groups, each consisting of 15 larvae. These groups were then incubated at 37 °C for 24 h. Following incubation, 5 μL of the respective treatments was injected directly into the larval hemocoel, between the third and fourth abdominal segments, using a Hamilton syringe.^19^ The experimental groups were as follows: Group 1: PBS only (negative control); Group 2: ZnPT (MIC 64 mg/L); Group 3: NYS (MIC 32 mg/L); Group 4: Combination of ZnPT (MIC 1 mg/L) + NYS (MIC 2 mg/L). *T. molitor* larvae were kept at 37 °C, and live larvae (responsive to touch) were recorded every 12 h for 72 h. Experiments were performed in three independent biological replicates.

### Statistical Analysis

All experiments were performed with at least three independent biological replicates. Data are presented as mean ± standard deviation (SD). Statistical analyses were performed using GraphPad Prism 8 software. The statistical tests applied to each experiment are specified in the corresponding figure legends. Comparisons between two groups were analyzed using an unpaired two-tailed *t* test when data met parametric assumptions, or the Mann–Whitney U test when normality assumptions were not met. Comparisons involving more than two groups were analyzed using one-way ANOVA followed by Tukey’s multiple-comparison test. Data distribution was assessed for normality prior to statistical testing. Statistical significance is indicated in the figures as *p* values: ns (not significant), *p* < 0.05, *p* < 0.01, *p* < 0.001, *p* < 0.0001.

## Supplementary Material





## Notes

Minimum inhibitory concentration of zinc pyrithione and nystatin in the presence or absence of sorbitol (Table S1); minimum inhibitory concentration of zinc pyrithione and nystatin in the presence or absence of promethazine (Table S2); synergistic activity of zinc pyrithione in combination with nystatin against *C. auris* assessed by dose–response matrix and synergy models (Figure S1); *in vitro* antibiofilm activity of zinc pyrithione, nystatin, and their combination against *C. auris* (Figure S2); cell membrane permeability analysis of *C. auris* following isolated and combined treatments with zinc pyrithione and nystatin (Figure S3); nucleotide leakage from *C. auris* after exposure to zinc pyrithione, nystatin, and their combination (Figure S4) (PDF)
Table of combinations, drugs, concentrations, and inhibition (XLSX)
