# Supplementary material for: Synergistic Antifungal Activity of Zinc Pyrithione and Nystatin against Multi-Drug-Resistant Candida (Candidozyma) auris: Evidence from In Vitro and In Vivo Models
Source: ACS Infect Dis. 2026 Feb 26;12(3):1064–74. doi: 10.1021/acsinfecdis.5c00822 (PMC12993841; doi:10.1021/acsinfecdis.5c00822)
Supplement: Supplementary file 1 [file id5c00822_si_001.pdf]

Supporting Information

Synergistic Antifungal Activity of Zinc Pyrithione and Nystatin Against Multidrug-Resistant *Candida* (*Candidozyma*) *auris*: Evidence from *In Vitro* and *In Vivo* Models

Larissa Rodrigues Pimentel,† Fabiola Lucini,† Ludmilla Cardoso Coferri, Yasmim Isabel Retore, Julia Pimentel Arantes, Cleison da Rocha Leite, Adriana Araújo de Almeida-Apolonio, Carlos Reinier Garcia Cardoso, Alex Polatto Carvalho, Fabricio Fagundes Pereira, Simone Simionatto, Kelly Mari Pires de Oliveira and Luana Rossato*

Larissa Rodrigues Pimentel - Health Sciences Research Laboratory, Federal University of Grande Dourados, Dourados, Mato Grosso do Sul 79804-970, Brazil; orcid.org/0009-0001-8613-3108; Email: larissa_pimentel05@hotmail.com

Fabiola Lucini - Health Sciences Research Laboratory, Federal University of Grande Dourados, Dourados, Mato Grosso do Sul 79804-970, Brazil; orcid.org/0000-0001-6658-4043; Email: fabiolalucini10@gmail.com

Ludmilla Cardoso Coferri - Health Sciences Research Laboratory, Federal University of Grande Dourados, Dourados, Mato Grosso do Sul 79804-970, Brazil; orcid.org/0009-0008-5196-6477; Email: ludmillacardosocoferri@gmail.com

Yasmim Isabel Retore - Health Sciences Research Laboratory, Federal University of Grande Dourados, Dourados, Mato Grosso do Sul 79804-970, Brazil; orcid.org/0009-0007-1716-465X; Email: yasiretore@gmail.com

Julia Pimentel Arantes - Health Sciences Research Laboratory, Federal University of Grande Dourados, Dourados, Mato Grosso do Sul 79804-970, Brazil; orcid.org/0000-0002-9089-4057; Email: julia_pimentel@live.com

Cleison da Rocha Leite - Microbiological Assays Laboratory, Federal University of Grande Dourados, Dourados, Mato Grosso do Sul 79804-970, Brazil; orcid.org/0009-0008-0531-1808; Email: cleisonleite38@gmail.com

Adriana Araújo de Almeida-Apolonio - Microbiological Assays Laboratory, Federal University of Grande Dourados, Dourados, Mato Grosso do Sul 79804-970, Brazil; orcid.org/0000-0002-3836-8519; Email: aaraujo.a@hotmail.com

Carlos Reinier Garcia Cardoso - Insect Biological Control Laboratory, Federal University of Grande Dourados, Dourados, Mato Grosso do Sul 79804-970, Brazil; orcid.org/0000-0002-2891-4475; Email: cr.garcia.cardoso@gmail.com

Alex Polatto Carvalho- Insect Biological Control Laboratory, Federal University of Grande Dourados, Dourados, Mato Grosso do Sul 79804-970, Brazil; Email: orcid.org/0000-0001-6989-4135; alexpolatto@hotmail.com

Fabricio Fagundes Pereira - Insect Biological Control Laboratory, Federal University of Grande Dourados, Dourados, Mato Grosso do Sul 79804-970, Brazil; orcid.org/0000-0003-1638-7409; Email: fabriciofagundes@ufgd.edu.br

Simone Simionatto - Health Sciences Research Laboratory, Federal University of Grande Dourados, Dourados, Mato Grosso do Sul 79804-970, Brazil; orcid.org/0000-0003-2367-0915; Email: simonesimionatto@ufgd.edu.br

Kelly Mari Pires de Oliveira - Microbiological Assays Laboratory, Federal University of Grande Dourados, Dourados, Mato Grosso do Sul 79804-970, Brazil; orcid.org/0000-0002-9897-7770; Email: kellyoliveira@ufgd.edu.br

Corresponding author

Luana Rossato - Health Sciences Research Laboratory, Federal University of Grande Dourados, Dourados, Mato Grosso do Sul 79804-970, Brazil; orcid.org/0000-0002-6115-3313; Email: luanarossato@ufgd.edu.br

[†] These authors have contributed equally to this work and share first authorship.

Table S1. Minimum Inhibitory Concentration of ZnPT and NYS in the presence or absence of sorbitol.

Treatment	ZnPT (mg/L)								NYS (mg/L)								MCF			
	24 hours				48 hours				24 hours				48 hours				24 hours		48 hours	
	MICi		MICc		MICi		MICc		MICi		MICc		MICi		MICc		MICi		MICi	
Strain	S-	S+	S-	S+	S-	S+	S-	S+	S-	S+	S-	S+	S-	S+	S-	S+	S-	S+	S-	S+
C. auris	64	≥256	1	2	64	≥256	1	16	32	16	2	2	32	32	2	16	≤0,01	0,06	0,5	≥8

S-: Absence of sorbitol, S+: Presence of sorbitol, MICi: Minimum Inhibitory Concentration (isolated), MICc: Minimum Inhibitory Concentration (combined), ZnPT: zinc pyrithione, NYS: nystatin, MCF: micafungin.

Table S2. Minimum Inhibitory Concentration of ZnPT and NYS in the presence or absence of promethazine.

Treatment	ZnPT (mg/L)								NYS (mg/L)								
	24 hours				48 hours				24 hours				48 hours				
	MICi		MICc		MICi		MICc		MICi		MICc		MICi		MICc		MIC
Strain	P-	P+	P-	P+	P-	P+	P-	P+	P-	P+	P-	P+	P-	P+	P-	P+	P
C. auris	64	0.06	1	0.01	64	0.06	1	1	32	1	2	0.01	32	8	2	1	>128

P-: Absence of promethazine, P+: Presence of promethazine, P: Promethazine, MICi: Minimum Inhibitory Concentration (isolated), MICc: Minimum Inhibitory Concentration (combined), ZnPT: zinc pyrithione, NYS: nystatin. MIC values represent results from three independent biological replicates.

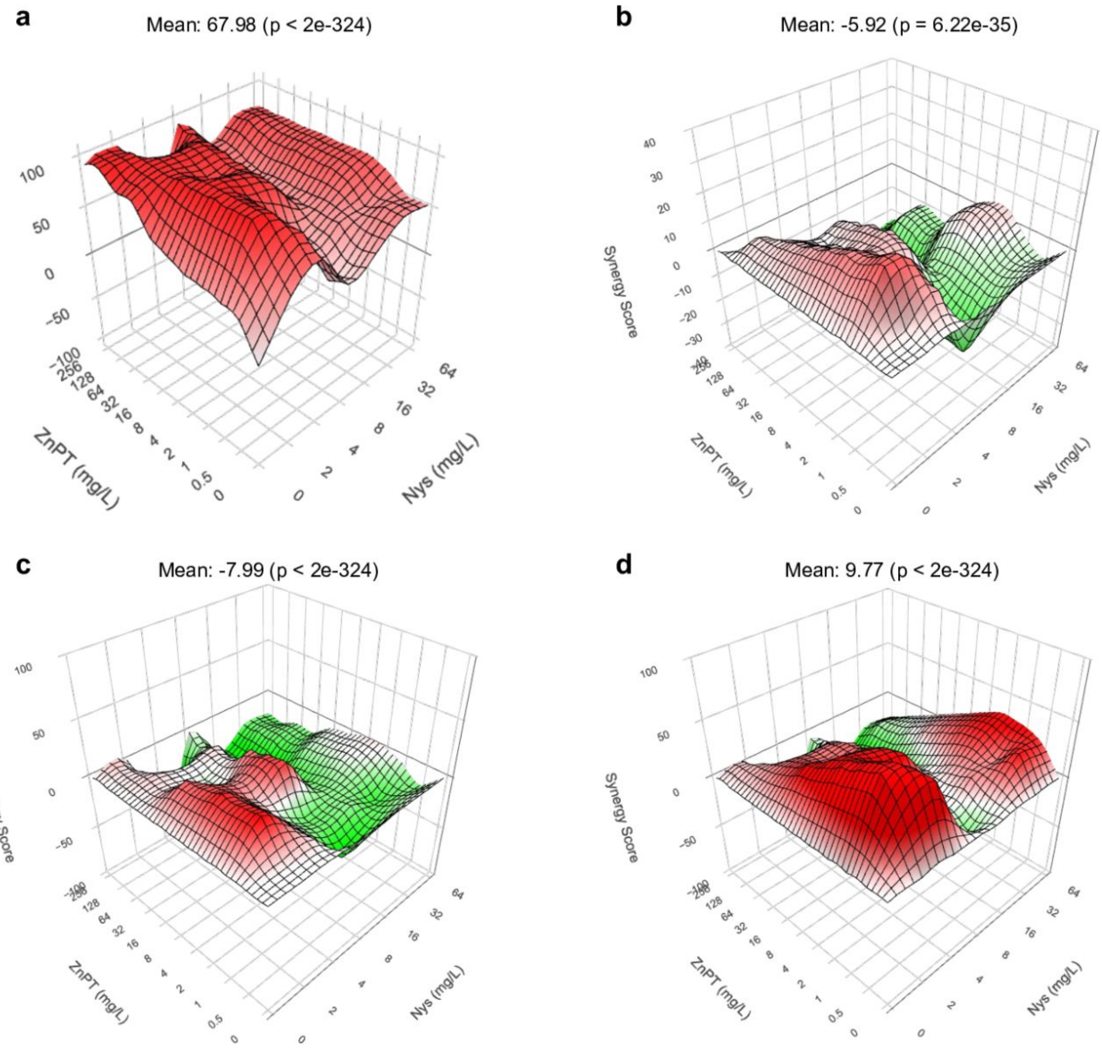

Figure S1. Synergistic activity of ZnPT in combination with NYS against C. auris (*in vitro*). **(a)** Dose–response matrix (inhibition). **(b)** ZIP synergy score surface plots. **(c)** Bliss independence synergy surface plot. **(d)** Loewe additivity synergy surface plot. For all synergy models, synergy scores >10 indicate synergism (red regions), scores between −10 and 10 indicate additive effects, and scores <−10 indicate antagonism (green regions). Shaded areas represent confidence intervals calculated internally by the SynergyFinder software. The corresponding X- and Y-axes indicate the concentration ranges encompassing the regions of greatest synergy. ZnPT: zinc pyrithione; NYS: nystatin. Checkerboard assays were performed in three independent biological replicates (See Table S3).

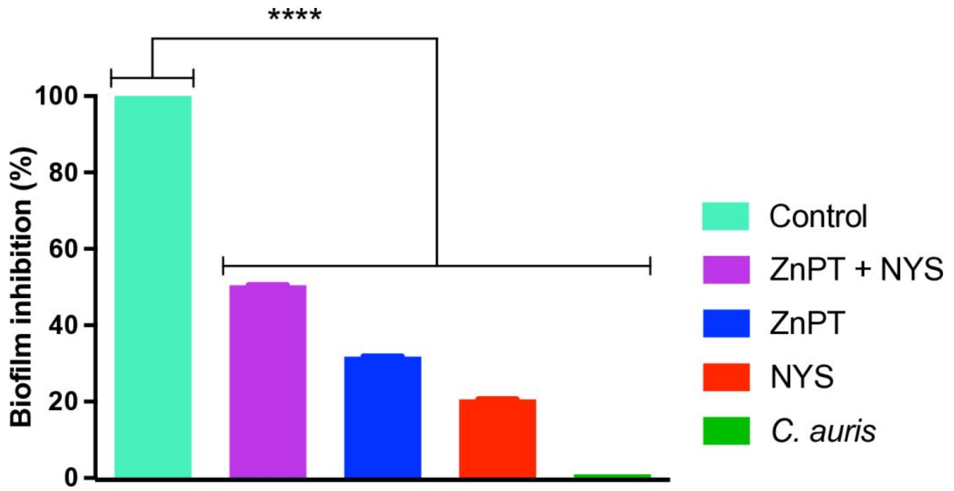

Figure S2. *In vitro* antibiofilm activity. The test was conducted at concentrations of ZnPT (at MIC 64 mg/L), NYS (at MIC 32 mg/L), ZnPT (at FIC 1 mg/L) + NYS (at 2 mg/L). The control indicates the absence of yeast, implying no biofilm formation. ZnPT: zinc pyrithione, NYS: nystatin. Data represent mean ± SD from three independent biological experiments. Statistical analysis was performed using one-way ANOVA with Tukey's multiple-comparison test. **** $p < 0.0001$.

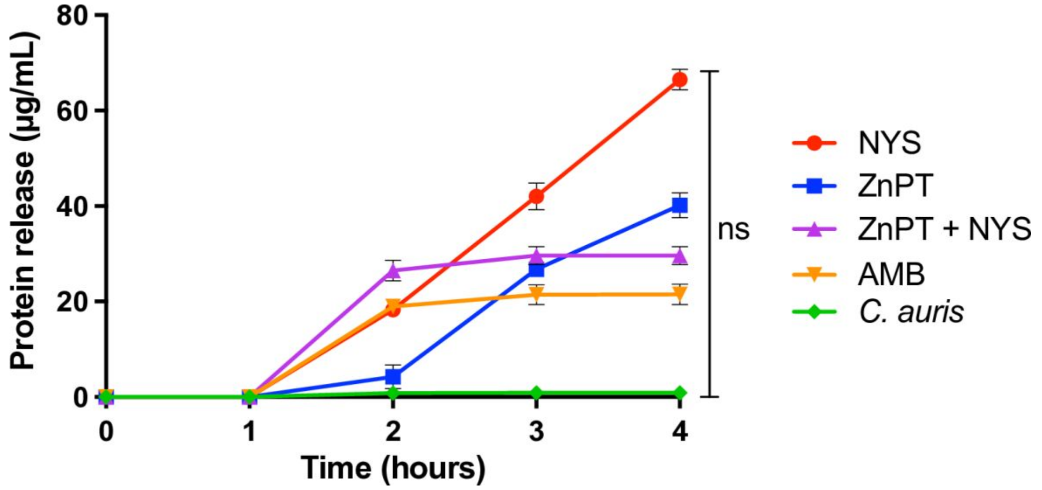

Figure S3. *In vitro* cell membrane permeability assay for *C. auris* following exposure to ZnPT, NYS, and their combination. Protein leakage was quantified using the BCA assay after treatment with ZnPT (at MIC 64 mg/L), NYS (at MIC 32 mg/L), and ZnPT (at FIC 1 mg/L) + NYS (at FIC 2 mg/L). AMB was used as a negative control for leakage, while untreated *C. auris* served as the positive control. Data are presented as mean ± SD from n = 3 independent experiments. A trend toward increased protein leakage was observed with the combination treatment, but statistical analysis revealed no significant (ns) differences compared to the positive control (*C. auris* alone). *ns* indicates no significant difference between the treatments and the positive control. Statistical analysis was performed using one-way ANOVA with Tukey's multiple-comparison test.

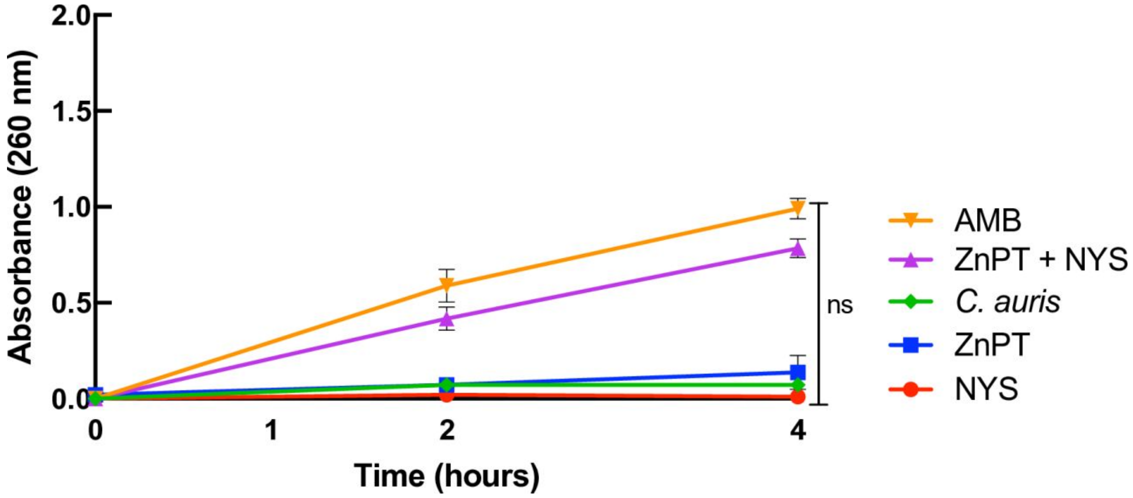

Figure S4. Nucleotide leakage from *C. auris* following isolated and combined treatments with ZnPT and NYS. Absorbance at 260 nm was measured in supernatants to assess nucleotide release after exposure to ZnPT (at MIC 64 mg/L), NYS (at MIC 32 mg/L), or the combination of ZnPT (at FIC 1 mg/L) + NYS (at FIC 2 mg/L). AMB was used as a positive control for nucleotide leakage, while untreated *C. auris* served as the negative control. Data shown as mean ± SD from n = 3 independent experiments. No significant (ns) differences were observed between the treatments and the positive control (AMB) at the 2-hour time point. At 4 hours, the combination treatment resulted in 0.75 mg/L nucleotide leakage, compared to 0.95 mg/L for AMB. Although a trend toward increased nucleotide leakage was observed with the combination treatment at 4 hours, statistical analysis revealed no significant differences between treatments. *ns* indicates no significant difference compared to the positive control (*C. auris* alone). Statistical analysis was performed using one-way ANOVA with Tukey's multiple-comparison test.
